# Postantifungal Effect of Micafungin against the Species Complexes of *Candida albicans* and *Candida parapsilosis*

**DOI:** 10.1371/journal.pone.0132730

**Published:** 2015-07-13

**Authors:** Sandra Gil-Alonso, Nerea Jauregizar, Elena Eraso, Guillermo Quindós

**Affiliations:** 1 Departamento de Inmunología, Microbiología y Parasitología, Unidad de formación e investigación multidisciplinar “Microbios y Salud” (UFI 11/25), Facultad de Medicina y Odontología, Universidad del País Vasco/Euskal Herriko Unibertsitatea, Bilbao, Bizkaia, España; 2 Departamento de Farmacología, Unidad de formación e investigación multidisciplinar “Microbios y Salud” (UFI 11/25), Facultad de Medicina y Odontología, Universidad del País Vasco/Euskal Herriko Unibertsitatea, Bilbao, Bizkaia, España; Louisiana State University, UNITED STATES

## Abstract

Micafungin is an effective antifungal agent useful for the therapy of invasive candidiasis. *Candida albicans* is the most common cause of invasive candidiasis; however, infections due to non-*C*. *albicans* species, such as *Candida parapsilosis*, are rising. Killing and postantifungal effects (PAFE) are important factors in both dose interval choice and infection outcome. The aim of this study was to determinate the micafungin PAFE against 7 *C*. *albicans* strains, 5 *Candida dubliniensis*, 2 *Candida Africana*, 3 *C*. *parapsilosis*, 2 *Candida metapsilosis* and 2 *Candida orthopsilosis*. For PAFE studies, cells were exposed to micafungin for 1 h at concentrations ranging from 0.12 to 8 μg/ml. Time-kill experiments (TK) were conducted at the same concentrations. Samples were removed at each time point (0-48 h) and viable counts determined. Micafungin (2 μg/ml) was fungicidal (≥ 3 log_10_ reduction) in TK against 5 out of 14 (36%) strains of *C*. *albicans* complex. In PAFE experiments, fungicidal endpoint was achieved against 2 out of 14 strains (14%). In TK against *C*. *parapsilosis*, 8 μg/ml of micafungin turned out to be fungicidal against 4 out 7 (57%) strains. Conversely, fungicidal endpoint was not achieved in PAFE studies. PAFE results for *C*. *albicans* complex (41.83 ± 2.18 h) differed from *C*. *parapsilosis* complex (8.07 ± 4.2 h) at the highest tested concentration of micafungin. In conclusion, micafungin showed significant differences in PAFE against *C*. *albicans* and *C*. *parapsilosis* complexes, being PAFE for the *C*. *albicans* complex longer than for the *C*. *parapsilosis* complex.

## Introduction

Invasive candidiasis is a leading cause of mortality worldwide, being *Candida albicans* the predominant cause of candidemia and invasive candidiasis. However, candidiasis due to non-*C*. *albicans* species, such as *Candida parapsilosis*, *Candida glabrata*, *Candida tropicalis*, *Candida krusei*, *Candida lusitaniae*, *Candida guilliermondii*, are increasing. Some of these species exhibit resistance or reduced susceptibility to fluconazole and other triazoles, echinocandins or amphotericin B. *C*. *parapsilosis* is associated to infections in neonates and young adults, usually related to the presence of central venous catheter and hyperalimentation [1]. *C*. *parapsilosis* is usually susceptible to most antifungal agents, but there are reports of infections caused by isolates with decreased susceptibility to azoles and echinocandins [2]. Molecular identification methods have unveiled new cryptic species within *C*. *albicans* and *C*. *parapsilosis* species complexes, such as *Candida dubliniensis* and *Candida africana* within the *C*. *albicans* complex or *Candida metapsilosis* and *Candida orthopsilosis* within *C*. *parapsilosis* complex. These cryptic species show differences in antifungal susceptibility and virulence, being their epidemiology and antifungal susceptibility a matter of increased interest [3–5].

Micafungin inhibits the synthesis of 1,3-β-D-glucan, an essential molecule of many pathogenic fungi wall architecture, and exhibits an excellent activity against a great number of *Candida* species many resistant to azoles [6]. Thus, micafungin is a very useful drug for the first line therapy of invasive candidiasis [7].

Postantifungal effect (PAFE) allows for sustained killing of fungus when it is exposed briefly to an antifungal, being a concentration-dependent process [8]. The existence of PAFE depends on both the fungal species and the class of the antifungal drug. Whereas antifungal drugs that have long PAFE may be given less frequently, the antifungal drugs with short PAFE may require a frequent administration [9]. For this reason, the PAFE may have a main clinical relevance in the design of dosing regimens for antifungal agents, such as micafungin. The PAFE of micafungin against various species of *Candida* has been evaluated in a few studies [10–13]. The aim of this study was to determinate the PAFE of micafungin against the species inside of the *C albicans* and *C parapsilosis* complexes.

## Materials and Methods

### Microorganisms

A total of 21 *Candida* strains were selected for testing: 14 strains from the *C*. *albicans* complex (*C*. *albicans*: 5 blood isolates [UPV/EHU 99–101, 99–102, 99–103, 99–104 and 99–105] and 2 reference strains [NCPF 3153 and 3156]; *C*. *dubliniensis*: 4 blood isolates [UPV/EHU 00–131, 00–132, 00–133, 00–135] and 1 reference strain [NCPF 3949]; *C*. *africana*: 1 vaginal isolate [UPV/EHU 97–135] and 1 reference strain [ATCC 2669]) and 7 strains from the *C*. *parapsilosis* complex (*C*. *parapsilosis sensu stricto*: 1 blood isolate [UPV/EHU 09–378] and 2 reference strains [ATCC 22019 and ATCC 90018]; *C*. *metapsilosis*: 1 blood isolate [UPV/EHU 07–045] and 1 reference strain [ATCC 96143]; *C*. *orthopsilosis*: 1 blood isolate [UPV/EHU 07–035] and 1 reference strain [ATCC 96139]). Fungal isolates were obtained from the culture collection of the Laboratorio de Micología Médica, Universidad del País Vasco/Euskal Herriko Unibertsitatea (UPV/EHU), Bilbao, Spain. Isolates were identified by their metabolic properties using the ATB ID 32C method (bioMérieux, Marcy l’Étoile, France) and by molecular methods, as previously described [14,15].

### Antifungal Agents

Micafungin (Astellas Pharma, Madrid, Spain) was dissolved in dimethyl sulfoxide (DMSO), to obtain a stock solution of 5120 μg/ml. The dilutions were prepared in RPMI 1640 medium with L-glutamine, 0.2% glucose and without NaHCO_2_ buffered to pH 7 with 0.165 M morpholinepropanesulfonic acid (MOPS) (Sigma-Aldrich, Madrid, Spain). Stock solutions were stored at – 80°C until use.

### In Vitro Susceptibility Testing

MICs, defined as minimum concentrations that produce ≥50 growth reduction, were determined following M27-A3 and M27-A3 S4 documents [16,17]. All MICs were measured in RPMI 1640 medium buffered to pH 7.0 with 0.165 M MOPS and results were read after 24 h of incubation.

### Time-Kill Procedures

Time-kill studies (TK) were performed as previously described [18–20]. Strains were subcultured on Sabouraud dextrose agar (SDA) plates prior to testing. Cell suspensions were prepared in sterile water by picking 3 to 5 colonies from a 24 h culture and the resulting suspension was prepared at 1 McFarland (≈ 10^6^ CFU/ml). One milliliter of the cell suspension was added to vials containing 9 ml of RPMI. TK were carried out on microtiter plates for the BioScreen C computer-controlled microbiological incubator (BioScreen C MBR, LabSystems, Helsinki, Finland) in RPMI (final volume 200 μl) by using an inoculum of 1–5 x 10^5^ CFU/ml. On the basis of MICs, micafungin concentrations tested were 0.12, 0.5 and 2 μg/ml for the *C*. *albicans* complex and 0.25, 2 and 8 μg/ml for the *C*. *parapsilosis* complex. These micafungin concentrations are achieved in serum after standard therapeutic doses [21]. Inoculated plates were incubated 48 h at 36 ± 1°C (30 ± 1°C for *C*. *africana*). At predetermined time points (0, 2, 4, 6, 24, and 48 h), 10 μl (0–6 h) or 6 μl (24–48 h) were collected from each culture well (control and test solution wells), serially diluted in phosphate buffered saline (PBS) and aliquots plated onto SDA. The lower limit of accurate and reproducible detectable colony forming units (CFU) counts was 200 CFU/ml. When the CFUs were expected to be less than 200 per milliliter, samples of 5 μl were taken directly from the test solution and plated. After incubation of the plates at 36 ± 1°C for 48 h (30 ± 1°C for *C*. *africana*), *Candida* colonies were counted. Each experiment was performed twice for each isolate. Plots of averaged colony counts (log10 CFU/ml) versus time were constructed and compared against a growth control (in the absence of drug). Also the antifungal carryover effect was determined as formerly reported [22].

### PAFE

PAFE studies were performed as described previously with slight differences [23]. Standard 1 McFarland turbidity cell suspensions were prepared in sterile distilled water, from which 1 ml was added to 9 ml of RPMI. Micafungin concentrations were the same as described for the TK. Following an incubation of 1 h, micafungin was removed by a process of 3 cycles of repeated centrifugations (2000 rpm, 10 min) and washed with PBS. After the final centrifugation, the fungal pellet was suspended in 600 μl of RPMI. All samples were incubated on microtiter plates for the BioScreen C at 36 ± 1°C, with a final volume of 200 μl. At the same predetermined time points described for the TK, samples were serially diluted in PBS and inoculated onto a SDA plate for CFU counting. When the colony counts were expected to be less than 200 CFU/mL, samples of 5 μl were taken directly from the test solution and plated. After incubation of the plates at 36 ± 1°C for 48 h, *Candida* colonies were counted. The lower limit of accurate and reproducible detectable colony counts was 200 CFU/ml. PAFE was calculated for each isolate as the difference in time required for control (in the absence of drug) and treated isolates to grow 1 log_10_ following drug removal. PAFE was also determined using the following equation: PAFE = T-C, where T = time required for counts in treated cultures to increase by 1 log_10_ unit above that seen following drug removal and C = time required for counts in control to increase by 1 log_10_ unit above that following the last washing.

### PAFE and TK data comparison

Fungicidal activity was described as a ≥ 3 log 10 (99.9%) reduction, and fungistatic activity was defined as a < 99.9% reduction in CFU from the starting inoculum size [24]. Plots of averaged colony counts (log10 CFU per milliliter) versus time were constructed and compared against a growth control. The ratios of the log killing during PAFE experiments to the log killing during time kill experiments were calculated. Time-kill and PAFE experiments were performed simultaneously.

### Statistical Analysis

Analysis of variance was performed to determine significant differences in PAFE (in hours) among species and concentrations, using GraphPad Prism 5.01 (GraphPad Software, San Diego, CA; USA). A *p* value < 0.05 was considered significant.

## Results

No antifungal carryover effect was detected in TK. Micafungin MICs for isolates from *C*. *albicans* and *C*. *parapsilosis* complexes are shown in Table 1.

**Table 1:** Micafungin MICs for *C*. *albicans* and *C*. *parapsilosis* complex strains.

Strain	MIC (μg/ml)
*C*.* albicans* NCPF 3153	0.25
*C*.* albicans* NCPF 3156	0.12
*C*.* albicans* UPV/EHU 99–101	0.25
*C*.* albicans* UPV/EHU 99–102	0.25
*C*.* albicans* UPV/EHU 99–103	0.12
*C*.* albicans* UPV/EHU 99–104	0.25
*C*.* albicans* UPV/EHU 99–105	0.12
*C*.* dubliniensis* NCPF 3949	0.25
*C*.* dubliniensis* UPV/EHU 00–131	0.25
*C*.* dubliniensis* UPV/EHU 00–132	0.12
*C*.* dubliniensis* UPV/EHU 00–133	0.12
*C*.* dubliniensis* UPV/EHU 00–135	0.06
*C*.* africana* UPV/EHU 97–135	0.12
*C*.* africana* ATCC 2669	0.06
*C*. *parapsilosis* ATCC 22019	2
*C*. *parapsilosis* ATCC 90018	1
*C*. *parapsilosis* UPV/EHU 09–378	2
*C*. *metapsilosis* ATCC 96143	2
*C*. *metapsilosis* UPV/EHU 07–045	2
*C*. *orthopsilosis* ATCC 96139	1
*C*. *orthopsilosis* UPV/EHU 07–035	1

The results of TK and PAFE experiments for *C*. *albicans*, *C*. *dubliniensis* and *C*. *africana* are shown in Table 2. Micafungin showed prolonged PAFE (≥ 37.5 h) against all strains of *C*. *albicans* complex with 2 μg/ml (*p* < 0.0001). With one of these strains (UPV/EHU 99–101) PAFE was > 43 h with 0.5 μg/ml. During TK tests, micafungin was fungicidal against 5 out of 14 (36%) strains of *C*. *albicans* complex (*C*. *albicans* NCPF 3156, UPV/EHU 99–101, 99–102, 99–105 and *C*. *dubliniensis* UPV/EHU 00–135). The extent of micafungin log-killing in TK ranged from 0.08 to 5.22 log at 2 μg/ml. After micafungin removal in PAFE experiments, fungicidal endpoint was achieved against 2 out of 14 (14%) strains of *C*. *albicans* complex (*C*. *albicans* UPV/EHU 99–102 and *C*. *dubliniensis* UPV/EHU 00–135). Moreover, the extent of killing during PAFE experiments ranged from 0.28 to 4.67 log with 2 μg/ml.

**Table 2:** PAFE results for *C*. *albicans* complex.

Isolate	Micafungin (μg/ml)	Killing (log)		PAFE/TK^1^	PAFE (h)
		TK	PAFE		
*C*. *albicans* NCPF 3153	0.12	0.21	NA^2^		0
	0.5	0.38	NA		0
	2	0.08	0.28	100	> 44
*C*. *albicans* NCPF 3156	0.12	NA	NA		0
	0.5	1.49	NA		0
	2	5.07	1.55	0	> 42
*C*. *albicans* UPV/EHU 99–101	0.12	2.25	0.56	2.04	0
	0.5	2.85	1.58	5.37	> 43
	2	5.1	1.81	0	> 43
*C*. *albicans* UPV/EHU 99–102	0.12	1.54	0.65	12.89	2.4
	0.5	2.26	0.42	1.44	0
	2	5	4.67	46.77	> 39.46
*C*. *albicans* UPV/EHU 99–103	0.12	NA	NA		0
	0.5	NA	NA		0
	2	1.49	0.52	10.68	> 44
*C*. *albicans* UPV/EHU 99–104	0.12	NA	0.27		0
	0.5	NA	0.02		0
	2	0.46	0.63	100	> 42
*C*. *albicans* UPV/EHU 99–105	0.12	0.63	0.62	98	0
	0.5	2.62	0.52	0.8	0
	2	5.22	0.55	0	> 42
*C*. *dubliniensis* NCPF 3949	0.12	0.12	0.04	82.56	0
	0.5	NA	0.11		0
	2	0.5	0.21	51.27	> 42
*C*. *dubliniensis* UPV/EHU 00–131	0.12	NA	NA		0
	0.5	NA	NA		20
	2	0.43	NA		44
*C*. *dubliniensis* UPV/EHU 00–132	0.12	NA	NA		0
	0.5	NA	NA		0
	2	0.51	NA		> 44
*C*. *dubliniensis* UPV/EHU 00–133	0.12	NA	NA		0
	0.5	0.02	NA		0
	2	0.7	0.4	50.1	42
*C*. *dubliniensis* UPV/EHU 00–135	0.12	0.86	NA		0
	0.5	2.22	0.24	1.05	0
	2	5	4.67	46.77	> 42
*C*. *africana* ATCC 2669	0.12	0.1	0.12	100	0
	0.5	0.1	0.12	100	0
	2	0.19	0.28	100	> 37.7
*C*. *africana* UPV/EHU 97–135	0.12	0.12	0.01	77.27	0
	0.5	0.08	0.24	100	3
	2	0.46	0.58	100	> 37.5

^1^ Ratio of the log killing during PAFE experiments to the log killing during time-kill experiments.

^2^ NA, not applicable (without any reduction in colony counts compared with the starting inoculum).

The mean value of PAFE/TK ratio was 43.25 (with 2 μg/ml) for *C*. *albicans* complex. Against 4 out of 14 strains (29%), the PAFE/TK ratio of micafungin at the highest tested concentration was 100, indicating that 1-hour exposure to micafungin accounted for up to 100% of the overall killing observed during TK. Additionally, a ratio of 100 at concentrations ≤ 2 μg/m was observed for *C*. *africana* (Table 2).

Table 3 summarizes the results of time-kill and PAFE experiments for *C*. *parapsilosis*, *C*. *metapsilosis* and *C*. *orthopsilosis* at each micafungin concentration. During TK, micafungin at 8 μg/ml caused significant reductions from the starting inoculum of each strain, with a killing activity that ranged from 1.67 to 5.43 log. However, during PAFE experiments, 1-hour exposure of the strains to micafungin did not cause important reductions in colony counts. PAFE of micafungin ranged 3.8 to 15.7 h (with 8 μg/ml); the longest PAFE (15.7 h) was reached against *C*. *parapsilosis* UPV/EHU 09–378. Micafungin at 8 μg/ml demonstrated fungicidal activity in TK against 4 out 7 (57%) strains from *C*. *parapsilosis* complex (*C*. *parapsilosis* UPV/EHU 09–378, *C*. *metapsilosis* ATCC 96143, UPV/EHU 07–045 and *C*. *orthopsilosis* ATCC 96139). However, after micafungin removal in PAFE experiments, it was not reached fungicidal endpoint against any of the tested strains. The lack of similarity between TK and PAFE data was also detected in the mean PAFE/TK ratio of 0.49, with 8 μg/ml, suggesting that 1-hour exposure to micafungin accounted for only a 2% of the overall killing observed during time-kill experiments; only one strain, *C*. *parapsilosis* UPV/EHU 09–378, showed a ratio of 100, with 2 μg/ml (Table 3).

**Table 3:** PAFE results for *C*. *parapsilosis* complex.

Isolate	Micafungin (μg/ml)	Killing (log)		PAFE/TK^1^	PAFE (h)
		TK	PAFE		
*C*. *parapsilosis* ATCC 22019	0.25	NA^2^	NA		0
	2	NA	NA		0
	8	1.67	0.08	2.56	6
*C*. *parapsilosis* ATCC 90018	0.25	0.16	NA		0
	2	0.12	NA		0
	8	2.12	0.07	0.89	5.3
*C*. *parapsilosis* UPV/EHU 09–378	0.25	NA	NA		0
	2	0.07	0.31	100	0
	8	5.27	0.22	0	15.7
*C*. *metapsilosis* ATCC 96143	0.25	0.02	NA		0
	2	NA	NA		0
	8	5.42	NA		5.4
*C*. *metapsilosis* UPV/EHU 07–045	0.25	NA	0.03		0
	2	NA	NA		0
	8	5.24	0.11	0	9.3
*C*. *orthopsilosis* ATCC 96139	0.25	NA	NA		2
	2	NA	NA		2
	8	5.43	NA		11
*C*. *orthopsilosis* UPV/EHU 07–035	0.25	NA	NA		0
	2	NA	NA		0
	8	1.91	NA		3.8

^1^ Ratio of the log killing during PAFE experiments to the log killing during TK experiments.

^2^ NA, not applicable (without any reduction in colony counts compared with the starting inoculum).

**Fig 1:**
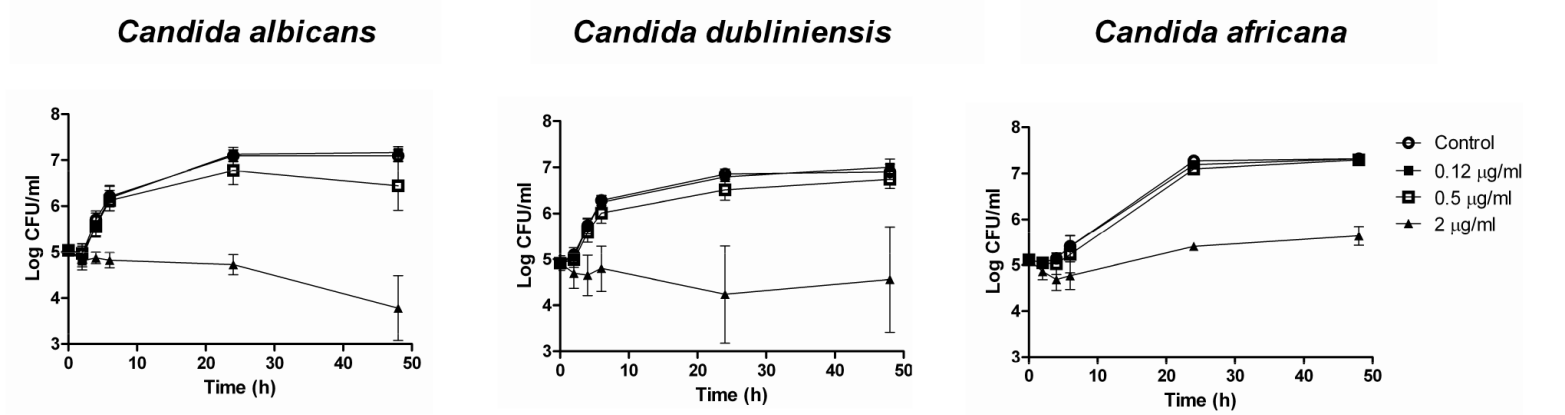
Mean TK curves from PAFE assays against 7 *C*. *albicans*, 5 *C*. *dubliniensis* and 2 *C*. *africana* strains. Each data point represents the mean result ± standard deviation (error bars). Open circles (○): control; filled squares (■): 0.12 μg/ml; open squares (□): 0.5 μg/ml; filled triangles (▲): 2 μg/ml.

**Fig 2:**
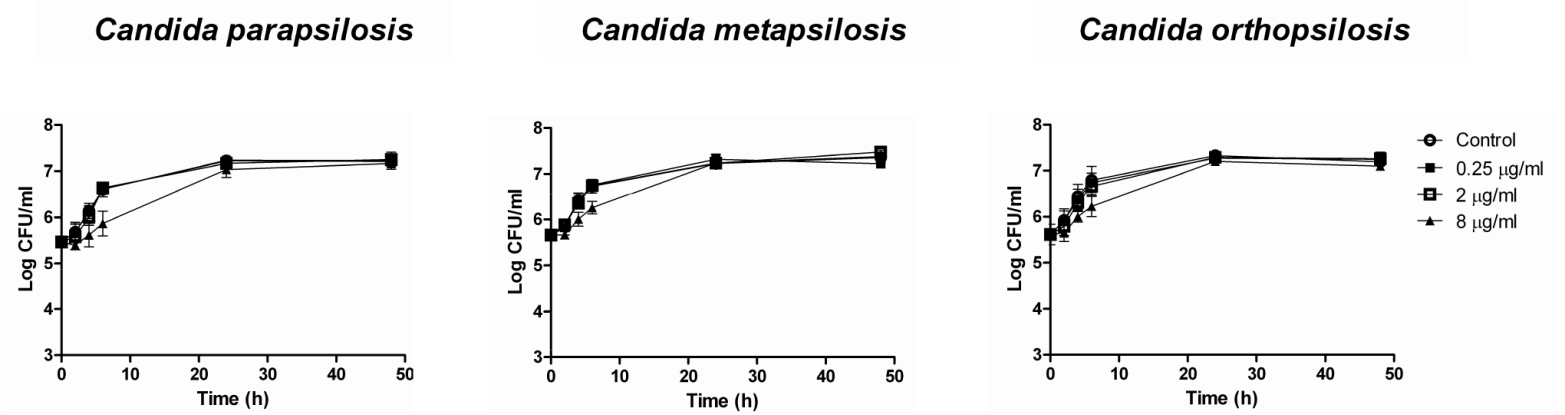
Mean TK curves from PAFE assays against 3 *C parapsilosis sensu stricto*, 2 *C*. *metapsilosis* and 2 *C*. *orthopsilosis* strains. Each data point represents the mean result ± standard deviation (error bars). Open circles (○): control; filled squares (■): 0.25 μg/ml; open squares (□): 2 μg/ml; filled triangles (▲): 8 μg/ml.

PAFE results for *C*. *albicans* complex (41.83 ± 2.18 h) differed from *C*. *parapsilosis* complex (8.07 ± 4.2 h) with the highest concentration of micafungin tested (*p* < 0.0001). This difference is also evident when comparing *C*. *albicans* and *C*. *parapsilosis* complexes curves from PAFE assays (Figs 1 and 2). Micafungin caused lethality (with 2 μg/ml) against *C*. *albicans* complex (Fig 1) that persisted during the 48 h testing period; however, in Fig 2 similar log (CFU/ml) slopes between micafungin and control can be observed.

## Discussion

TK and PAFE experiments of micafungin against *Candida* have usually included a low number of isolates [10–13]. This is the first study that has evaluated PAFE of micafungin against *C*. *dubliniensis*, *C*. *africana*, *C*. *metapsilosis* and *C*. *orthopsilosis*. *C*. *dubliniensis* and *C*. *africana* are cryptic species from *C*. *albicans*. Similarly *C*. *metapsilosis* and *C*. *orthopsilosis* are cryptic species from *C*. *parapsilosis*. These species have different in vitro susceptibility to antifungal agents [3,4,25]. Additionally, PAFE is an important factor in both dose interval choice and outcome.

MICs for *C*. *albicans* and *C*. *parapsilosis* complexes were consistent with other studies of micafungin activity in vitro against these species [26]. Moreover, we also found that micafungin reached fungicidal endpoint against 4 out of 7 strains of *C*. *albicans* (with 2 μg/ml) and against 1 out of 3 strains of *C*. *parapsilosis* (with 8 μg/ml), during TK experiments. This fungicidal activity has also been reported by Smith et al. [11] against both species.

After micafungin removal, Nguyen et al. [11] observed fungicidal activity against 1 out 4 strains of *C*. *albicans*, 1 out of 3 strains of *C*. *parapsilosis*, 2 out of 3 strains of *C*. *glabrata* and 1 out of 2 strains of *C*. *krusei* (with range concentrations 0.12 to 8 μg/ml). Similarly, in the current study, the fungicidal endpoint was reached against 1 out of 7 strains of *C*. *albicans* at the highest tested concentration (2 μg/ml). Nevertheless, after micafungin removal, no fungicidal endpoint was achieved against *C*. *parapsilosis* [11].

Micafungin (8 μg/ml) displayed PAFE against *C*. *parapsilosis* complex that ranged from 3.8 to 15.7 h, being the longest PAFE against *C*. *parapsilosis* UPV/EHU 09–378. These results are similar to previous reported by Smith et al. [10] Other authors have demonstrated that a short exposure (1 h) of *C*. *albicans* to low concentrations (0.125 to 1 μg/ml) of micafungin, resulted in a PAFE of 5 h [12]. Our current findings demonstrate that micafungin produced a longer PAFE against *C*. *albicans* than those previously reported, being the PAFE > 40 h with 2 μg/ml against all strains. Manavathu et al. [12] compared PAFE of different antifungal drugs against *C*. *albicans* and *Aspergillus fumigatus* and stated that antifungal drugs with fungicidal activity tend to possess longer PAFE than fungistatic ones. On the other hand, Ernst et al. [23] observed that fluconazole displayed no measurable PAFE against none of the studied microorganisms, while echinocandins displayed prolonged PAFE of greater than 12 h against *C*. *albicans* with concentrations ≤ 0.12 μg/ml. Our current findings differed from these ones, as no measurable PAFE was detected against *C*. *albicans* at such low micafungin concentrations (0.12 μg/ml) except for one strain, UPV/EHU 99–102. In order to investigate the effect of exposure time on the observed PAFE, Ernst et al. studied the PAFE of caspofungin and amphotericin B after 0.25, 0.5 and 1 h exposure times concluding that PAFE was not affected by the exposure time: 0.25 h exposure produced the same PAFE as 1 h exposure [23]. Similarly, Moriyama et al. reported that the maximum PAFE against *Candida* occurred with caspofungin exposures of 5 or 15 minutes [8]. As performed in other PAFE experiments, in which PAFE was determined after 1 h exposure [10–12], we have studied the PAFE of micafungin after 1 h exposure.

In another study, Ernst et al. also found PAFE with micafungin against *C*. *albicans*, *C*. *krusei*, *C*. *tropicalis* and *C*. *glabrata*, [13]. Micafungin and anidulafungin had greater activity than caspofungin, and none of the echinocandins depicted fungicidal activity against *C*. *parapsilosis*. However, the three echinocandins reached the fungicidal endpoint against *C*. *orthopsilosis* and *C*. *metapsilosis* [19]. Results from our study differ from these reports as we have found that micafungin was fungicidal only against one strain of *C*. *parapsilosis*.

Previous studies have evaluated PAFEs of anidulafungin and caspofungin against *Candida*, and have shown that anidulafungin achieved fungicidal activity against *C*. *parapsilosis*, but not against *C*. *albicans*, and caspofungin did not show fungicidal activity [27,28].

Our PAFE studies demonstrated that micafungin produced concentration-dependent, strain-dependent and complex-dependent antifungal activity following drug removal. PAFE was measurable at the higher concentration, and this effect was enhanced by increasing the concentration of the antifungal drug, with highest concentration resulting in the longest PAFE in each case. One of the most notable findings of this study was the PAFE of micafungin against *C*. *albicans* complex. Micafungin exerted prolonged PAFE against *C*. *albicans* complex, and 1 h exposure to micafungin accounted for up to 100% of the overall killing observed during TK experiments in 29% of the studied strains. The results are consistent with a rapid onset of anticandidal activity of micafungin, which might be explained by a rapid association with its target (1,3-β-D-glucan synthase). Alternatively, it has also been suggested that the drug, as a large lipopeptide with a fatty acid side chain, could rapidly intercalate with the phospholipid bilayer of the *Candida* cell membrane and subsequently access its target over time [29].

Recently, Ellepola et al. studied the PAFE of nystatin, amphotericin B, ketoconazole and fluconazole against oral *C*. *dubliniensis* isolates, concluding that nystatin, amphotericin B and ketoconazole produced a detectable PAFE, whereas fluconazole did no display any measurable PAFE [30,31]. This finding is consistent with previously published by Ernst et al. [23]. Kovács et al. reported caspofungin PAFE in 2 *C*. *albicans* strains [32].

In conclusion, micafungin showed significant differences in PAFE against *C*. *albicans* and *C*. *parapsilosis* complexes, being PAFE of micafungin for the *C*. *albicans* complex longer than against the *C*. *parapsilosis* complex. These differences in the PAFE could be explained by the distinct microorganism growth characteristics, the antifungal drug binding affinity to the targets, or differences in the amount of β-glucan in the fungal cell wall. These PAFE differences for *C*. *parapsilosis* and other *Candida* species might have important therapeutic implications. The current data could be useful in optimizing dosing regimens for micafungin against *C*. *albicans*, *C*. *dubliniensis*, *C*. *africana*, *C*. *parapsilosis*, *C*. *metapsilosis* and *C*. *orthopsilosis*. However, further animal studies and human clinical trials are needed to explore their potential clinical usefulness and applications.
